# Effects of White Noise Sound on the Severity of Muscle Vaccination Pain in Children Under Two Years

**DOI:** 10.22037/ijcn.v18i2.38746

**Published:** 2024-03-12

**Authors:** Abbas SHAMSALINIA, Zahra FOTOKIAN, Zahra Jannat ALIPOUR, Yadollah ZAHEDPASHA, Fatemeh MOHAMMADKHAH

**Affiliations:** 1 Nursing Care Research Center, Health Research Institute, Babol University of Medical Sciences, , Babol, Iran; 2 Non-Communicable Pediatric Disease Research Center, Health Research Institute , Babol University of Medical Sciences; , Babol, Iran.

**Keywords:** Infant, Pain, Vaccination, White Noise

## Abstract

**Objectives:**

Pain and its control is a significant health problem worldwide. The present study aimed to determine the effects of white noise (bird sound) on the severity of muscle vaccination pain in children under two years old.

**Materials & Methods:**

This study was a case-control study conducted in 2021. The samples included seventy children under two years old referred to the health centers in Ramsar City, Iran. The samples were selected using the convenient sampling method and divided into experimental and control groups. The data were collected using the demographic characteristics questionnaire, facial expression, and pain assessment in pediatric patients (FLACC). They were then analyzed by SPSS16 using an independent t-test and analysis of covariance (P<0.05).

**Results:**

A significant difference was observed between the severity of muscle vaccination pain in children in the two groups (p=0.042); the pain intensity mean in the experimental group (6.45±2.01) was lower than the control group (8.94±1.28).

**Conclusion:**

This method can be a harmless and inexpensive intervention to reduce pain intensity and behavioral pain responses in infants during painful procedures, especially vaccination.

## Introduction

Pain is an unpleasant feeling and a psychological experience caused by possible or actual injuries (1). Pain and its control is a significant health problem worldwide (2). In the past, infants were believed not to feel pain, but new studies have shown that an infant’s functional, autonomic, nervous, and chemical systems are developed enough to feel pain. Failure to relieve pain can lead to irritability, fatigue, and poor health. In addition, repeated experiences of pain in infancy can lead to relatively permanent changes in the autonomic nervous system. One of the most important clinical effects of the early experience of pain in infancy and childhood can be its destructive effects on the development of the nervous system, attention, and learning ability (3). Failure to relieve pain can lead to profound and long-term consequences and emotional stress at different levels. Pain relief is a basic need and right of all children, in which painful processes in children should be predicted, evaluated, and relieved (4). Pain is the fifth most vital sign, so healthcare providers should monitor pain with the same care and attention in controlling and recording vital signs (5). In this regard, adequate pain control in infants is of great importance. Today, infants are involved in different predictive, diagnostic, or treatment measures that cause different pain levels. Vaccination is the most common painful procedure that infants experience repeatedly due to the national immunization program. It is stressful for infants and affects their parents (6). Pain and emotional stress caused by the injections may cause abnormal reactions in the child and be one of the reasons for delaying the child’s vaccination. Therefore, any activity and measure to manage vaccination pain can be considered essential to health measures. However, pain management requires teamwork and a multidisciplinary approach. In this regard, as nurses play an exclusive role in the team and perform the pharmacological and non-pharmacological methods to reduce pain, they should evaluate the child’s behavioral responses to pain and its physiological aspects and use methods to minimize the experienced pain (7). 

Vaccination pain management measures include pharmacological and non-pharmacological treatments. Due to possible side effects, pharmacological treatments are not considered optimal pain control methods. Therefore, non-pharmacological treatments are of particular importance (6). Furthermore, recent concerns about the side effects of medications have led to a greater tendency to use non-pharmacological interventions to relieve the pain of diagnostic and therapeutic procedures (8). Implementing non-pharmacological methods for vaccination in health centers in the limited physical space faces some challenges. These methods can include the use of medications, ice (9), kangaroo care (skin-to-skin contact) (10), dextrose, sucrose, and oral glucose (4, 11-13), breastfeeding (4, 14), distraction (use of a rattle) (15), black pepper seeds (14), breastfeeding and placing the infant in mother’s arms (3), massaging Hugo point (an essential analgesic point of the body in the middle of the bisector of the angle between the first and second metacarpal bone), with and without ice (6), and listening to music (7). The American Academy of Pediatrics (2006) has outlined specific principles for minimizing pain in newborns, including avoiding painful stimuli, using non-pharmacological methods to prevent or alleviate pain, employing experienced personnel in the neonatal intensive care unit, and using valid and reliable pain assessment tools (7).

One of the non-pharmacological methods recently attracting patients’ and healthcare providers’ attention is the effect of white noise on alleviating pain. White noise is an effective method to alleviate vaccination pain. The goal of pain management in infants is to relieve pain and help infants cope with it (7). White noise contains all frequencies across the spectrum of audible sound equally, such as wind blowing through trees, waterfall sounds, or ocean waves (7). Other white noises include the sound of rain or birds (15). The researchers found no study on the effect of bird sounds on the severity of infants’ muscle vaccination pain; therefore, the present study sought to answer this question: Is the sound of birds effective in alleviating vaccination pain in children? Since bird sound is appropriate, simple, and inexpensive to relieve infants’ pain, it can reduce the severity of vaccination pain in children if confirmed. In this regard, the present study aimed to investigate the effect of white noise (sound of birds) on the severity of muscle vaccination pain in children under two years old. 

## Materials & Methods

This study was a clinical trial. It was conducted on seventy infants referred to the vaccination department of all health centers in Ramsar, Iran, in 2021. Under a letter of introduction from Babol University of Medical Sciences and after coordination with Sari University of Medical Sciences, Ramsar Health Network, and its head of vaccination department, the researcher was provided with a list of urban health centers in Ramsar and referred to these centers for sampling. The preliminary sampling method was convenient sampling. The inclusion criteria were as follows: Children aged two, four, and six months who were referred to the centers for pentavalent vaccine injection, being in the normal weight and height percentile as specified in health centers, having a normal temperature before the vaccine injection (below 37.5 °c), being calm, not taking acetaminophen or any other analgesic from the previous night, absence of hospitalization history, being breastfed at least two or three hours before the vaccine injection, absence of any insomnia history based on the information reported by the mother (6), good mental, psychological, and physical conditions of the mother, no hearing problem in the child, and healthy mothers with no deceased baby (self-report). The samples were divided into experimental and control groups using the random allocation method. After explaining the research purpose and method, the written informed consent form was obtained from the infants’ parents. Then, the infants were examined regarding height, weight, and temperature, and the demographic characteristics questionnaire was completed.

The parents were contacted and informed regarding not taking acetaminophen or other analgesics. In the experimental group, the white noise (sound of birds) started one minute before the vaccine injection, and infants listened to it for five minutes via headphones on the Samsung Galaxi j2 Core mobile phone. The sound of birds was played in mp3 format, 128 kbps, 7 megabytes, 31 decibels, playable on computers, mobile phones, cars, and other music players, downloaded from www.sarzamindownload.com. The control group received routine care for vaccination. The intramuscular injection was performed under the same conditions for both groups by a skilled and experienced researcher with 16 years of experience (3).

During vaccination, the infant was held by the infant’s companion and the vaccinator. For blinding, the lead researcher entered the vaccine room with an observer after the intervention and 7 seconds before the injection. All infants received their vaccination while lying on their backs on the vaccination bed (4, 16). The injection site was disinfected with alcohol-soaked cotton from the center of the injection site to the surrounding area. After 15 seconds, the vaccine was injected deep into the anterior outer part of the thigh with a syringe with a 23 gauge needle and a needle length of 2.5 cm manufactured by Farid Company under hygienic instructions. Attempts were made to provide a calm and suitable environment for all infants during vaccination. The severity of vaccination pain in infants of both groups was assessed through a behavioral pain scale (FLACC). The pain assessment time was 15 seconds after injection, and the highest level of reaction observed was considered the infant’s pain intensity score. The data collection tools used in this study were the demographic characteristics questionnaire and FLACC. 

The demographic characteristics questionnaire included infants’ age, gender, weight and height, parents’ education and occupation, birth rank, and breastfeeding time before vaccination [6]. This study assessed the pain in infants using a behavioral pain scale. This tool was designed by Voepel-Lewis et al. in 1997 and comprised five subscales: facial expression, leg movement, activity, cry, and consolability. Each subscale was scored between 0 and 2, and the total score of the questionnaire was between 0 and 10 (17). The scores were divided into four levels to classify the total score: easy (0 points), mild pain (1-3), moderate pain (4-6, and severe pain (7-10). In addition, the validity and reliability of the tool were investigated and confirmed by Voepel-Lewis et al. (17). Other studies, e.g., Sadeghi (18) and Babaei (19), confirmed the validity and reliability of the tool. The data were analyzed by SPSS using descriptive and analytical tests (independent t-test and analysis of covariance). After obtaining permission from the ethics committee of Babol University of Medical Sciences (IR.MUBABOL.REC.1400.003), explaining the research purposes, and obtaining the written informed consent forms [consent was obtained from all adult participants and the parents or legal guardians of minors], the data were collected. The researchers tried to address the confidentiality and trustworthiness.

## Results

Most of the experimental group (n=22) infants were male, and 21 infants in the control group were female. Other demographic characteristics of the two groups are presented in Table 1. No significant statistical difference was observed between the two groups regarding demographic characteristics (P>0.05).

Regarding the insignificant results of the Shapiro-Wilk test for research variables in both experimental and control groups, the independent t-test was used for intergroup comparison. Table 2 showed that the severity of muscle vaccination pain for children under two years old was high in both experimental (68.6%) and control (94.3%) groups. The chi-square test results showed a significant relationship in the severity of muscle vaccination pain in children under two years old between the two groups (P=0.042); the severity of muscle vaccination pain in the experimental group was lower than the control group (Table 2). 

According to the independent t-test, there was a significant difference between the severity of muscle vaccination pain in both experimental and control groups (P<0.001); the mean pain level in the experimental group (6.45±2.01) was lower than in the control group (8.94±1.28). The results were also presented in terms of subscales of pain severity. The results of the independent t-test showed that there was a significant difference in the subscales of leg movement (P<0.001), activity (P<0.001), cry (P=0.046), and consolability (P<0.001) between the two groups. The mean pain level in the experimental group was lower than that of the control group in leg movement, activity, cry, and consolability subscales. The results also showed no significant difference between the two groups in the children’s facial expressions regarding vaccination pain (P=0.140) (Table 3). 

Analysis of covariance test was used to investigate the effect of group (experimental and control) and demographic characteristics on the severity of muscle vaccination pain in children. The results of the analysis showed that the group variable was influential on the severity of pain during vaccination by modifying demographic characteristics; that is, there was a significant difference between the two groups in modified severity of pain, which was 41%, according to the effect size, and at an acceptable level. Moreover, the infants’ age significantly affected the severity of pain during vaccination (P=0.015). That is, as the age of infants increases, the severity of pain during vaccination decreases (Table 4).

**Table 1:** Demographic characteristics of the infants under study (n=70)

Variable	group	Experimental		Control		Statistic of the test	Significance level
	Classification	No.	Percentage	No.	Percentage		
Gender	girl	13	37.1	21	60	3.660 a	0.056
	boy	22	62.9	14	40		
Mother’s education	Below diploma	8	22.9	2	5.7	4.441 a	0.109
	Diploma	14	40	15	42.9		
	Academic	13	37.1	18	51.4		
Father’s education	Below diploma	12	34.3	7	20	2.786 a	0.248
	Diploma	17	48.6	17	48.6		
	Academic	6	17.1	11	31.4		
Mother’s occupation	Housewife	24	97.1	28	80	5.914 a	0.052
	Employee	0	0	5	14.3		
	Self-employed	1	2.9	2	5.7		
Father’s occupation	Worker	2	5.7	0	0	3.669 a	0.160
	Employee	3	8.6	7	20		
	Self-employed	30	85.7	28	80		
Taking acetaminophen the night before injection	No	35	100	35	100	1.014 a	0.314
	Yes	0	0	0	0		
Insomnia the night before injection	No	35	100	35	100	1.014 a	0.314
	Yes	0	0	0	0		
Infant’s age (month), mean(standard deviation)		4.17(1.70)		4.17(2.07)		0.000 b	1
Infant’s height (cm), mean(standard deviation)		63.77(6.38)		63.05(5.67)		0.494 b	0.623
Infant’s weight (kg), mean(standard deviation)		6.84(1.35)		6.85(1.84)		-0.022 b	0.983
Body temperature (centigrade), mean(standard deviation)		37.01(0.08)		37.11(0.32)		-1.773 b	0.081

a: chi-square test

b: independent t-test, p>0.05

**Table 2:** Comparison of muscle vaccination pain in children under 2 years old in both experimental and control groups(n=70)

Group	muscle vaccination pain Levels				Statistic	Significance level
	Easy	Mild	Moderate	Severe		
Experimental	1(2.9)	3(8.6)	7(20)	24(68.6)	8.199 a	0.042
Control	0(0)	0(0)	2(5.7)	33(94.3)		

Data were inserted based on the number (percentage), a: chi-square test, P<0.05

**Table 3:** Comparison of the severity of pain during vaccination in the experimental and control groups(n=70)

Variable	Group		Mean difference	Statistic	Significance level
	Experimental (mean ±standard deviation)	Control (mean ±standard deviation)			
Facial expression	0.49 ± 1.77	1.91±0.28	-0.14	-1.50 a	0.140
Leg movement	0.52 ± 1.11	1.74±0.44	-0.63	-5.39 a	<0.001
Activity	0.37 ± 0.91	1.57±0.50	-0.66	-6.22 a	<0.001
Cry	0.62± 1.71	1.94±0.23	-0.23	-2.04 a	0.046
Consolability	0.33 ± 0.94	1.77±0.42	-0.83	-9.02 a	<0.001
Total pain score	2.01 ± 6.45	8.94±1.28	-2.49	-6.18 a	<0.001

a: independent t-test

**Table 4:** The effect of group (experimental and control) and demographic characteristics on the severity of pain during intramuscular vaccination in children(n=70)

Variable	Freedom degree	Mean of squares	Statistic F	Sig. level	Effect size
Group (experimental and control)	1	98.98	37.09	<0.001	0.403
Gender	1	0.88	0.33	0.566	0.006
Mother’s education	2	4.13	1.54	0.222	0.053
Father’s education	2	1.97	0.73	0.482	0.026
Mother’s occupation	2	0.52	0.19	0.823	0.007
Father’s occupation	2	4.16	1.72	0.187	0.059
Infant’s age	1	17.01	6.37	0.015	0.104
Infant’s height	1	0.95	0.35	0.553	0.006
Infant’s weight	1	1.55	0.58	0.488	0.010
Infant’s body temperature	1	5.28	1.98	0.165	0.035
Error	55	2.66			

## Discussion

The present study aimed to investigate the effect of white noise (bird sounds) on the severity of muscle vaccination pain in children under two years old. According to the results, the muscle vaccination pain in both experimental and control groups was severe, while Kucukoglu et al. (2016) showed that the pain severity of children was medium in the experimental group but severe in the control group (7); this difference can be due to difference in age groups, type of white noise used in the experimental group, and the tools of assessment, despite the severity of pain in the control groups of both studies being high. 

Pain in children must be identified to prevent physical and mental problems. Failure to relieve pain in children is associated with increased anxiety, fear of health staff, and increased sensitivity to pain (20). Pain management is one of the essential criteria of care quality (21). Pain management and evaluation are part of nursing care, and nurses play a critical role (21). Vaccination is a painful process in childhood, which should be effectively dealt with to be relieved. 

The present study used white noise (bird sound) to relieve muscle vaccination pain in the children under study. The results showed that the severity of pain in the experimental group was lower than in the control group. Kucukoglu et al. (2016) (7), ERKAL AKSOY et al. (2019) (22), and Lin et al. (2020) (23) found similar results. White noise is a monotonous and continuous sound resembling the mother’s heartbeat (8). Infants are affected by the mother’s heartbeat and feel calm and relaxed after hearing a similar sound and rhythm after delivery (7). The mother’s voice is familiar to the baby from the womb, and they can differentiate their mother’s voice from other voices. The mother’s voice can stabilize the newborn’s state and relieve and alleviate the infant’s pain. The mother’s voice is the first and vital low-frequency sound for the fetus. Three-day-old infants can recognize the mother’s voice and heartbeat, positively affecting their physiological and behavioral responses (24). They can differentiate their mother’s voice from other familiar voices (25), and the fetus also has a considerable ability to understand sounds and learn to hear in the womb. Rand et al. (2014) conducted a study investigating the physiological responses to the mother’s voice and its persistence in very premature infants’ first month of life. The results showed that the mother’s voice reduces premature infants’ heart rate, improves autonomic stability, and provides a relaxing environment for these infants (26). Babaei et al. (2016) investigated the effect of a mother’s voice on pain after a pediatric tonsillectomy and showed that the mother’s voice alleviated the children’s pain (19). White noise (bird’s sounds) stimulates infants’ sense of hearing. Various studies have investigated sounds as an intervention to alleviate pain. In this regard, Motahedian et al. (2012) found that music has a considerable effect on relieving pain after surgical operation and reduces the use of narcotics (27).

On the other hand, white noise can also be used as a distraction, a non-pharmacological method to relieve pain. In this regard, Nazemzade et al. (2013) reported in their review study that implementing distraction techniques leads to a reduction in pain and anxiety caused by treatment procedures in children with different diseases (28). Gedam et al. (2013) investigated the effect of distraction techniques [sound-producing toys and cartoon movies] during immunization to reduce behavior response scores to pain in toddlers, and the results indicated a reduced severity of pain (29). On the other hand, white noise penetrating the level of consciousness causes distraction; the thalamus receives musical stimuli and transmits them to the brain through a network activation system, and all these affect the intelligence, memory, and perception of the person and causes distraction from pain. Music also affects the pituitary gland, causing the release of endorphins, which in turn relieves pain (30). White noise is a pleasant stimulus and a multi-sensory experience that creates various sensory stimuli through auditory, tactile (vibration), and muscular stimulation, which is hard to ignore and attracts the individual’s senses, distracting the person from the pain (31). Believingly, through distraction, the retinal formation of the brainstem is sufficiently stimulated by various sensory stimuli. Thus, it can selectively prevent and ignore the transmission of senses such as pain (32). Therefore, a variety of distraction techniques can be used to lessen the impact of the destructive experience of severe pain in children during painful procedures. 

Studies have investigated the effects of natural sounds, such as birds’ sounds, on reducing stress and improving psychological states (15) and mental health (33). Natural sounds improve cognitive processes (34). Various studies have investigated the effect of white noise on children’s pain and its effectiveness (7, 35-37). They have also suggested using white noise while performing painful procedures in infants to alleviate their pain. White noise intervention is environmental care to positively impact behavioral and physiological signs, such as decreasing heart rate, respiratory rate, blood pressure, oxygen consumption, and muscle contraction. (38). However, this result is inconsistent with the results of Arts et al. (1994), who compared the effect of music with anesthetic ointment and found that anesthetic ointment was an effective intervention to alleviate the pain of venipuncture in children. In contrast, the music was ineffective in alleviating the pain (39). It can be due to the differences in the selected white noise, the skill of performing the painful process, and the environment of intervention implementation. 

In the present study, the mean children’s pain severity in the subscales of leg movement, activity, cry, and consolability were lower in the experimental group. The children responded to painful stimuli and the stress they caused with behavioral signs such as crying, eyebrow-raising, and leg and arm movement, as well as physiological signs such as increased heart rate and decreased arterial blood oxygen saturation (3, 13). In the present study, since the muscle vaccination pain was lower in the experimental group than in the control group, the pain severity in the subscales of leg movement, activity, crying, and consolability in the experimental group was lower than in the control group. White noise is a distraction (bird sound) in reducing the severity of vaccination pain in children. Gedam et al. (2013) and Hadadi Moghadam et al. (2010) used the distraction method to reduce the severity of pain, and the results indicated a reduction in pain severity, changes in facial expression, body movement, and duration of crying in the experimental group (29, 32). Distraction is a method to alleviate pain, which can control pain in children by interfering with pain stimuli (40). The lack or absence of various sensory stimuli causes the person only to pay attention to the painful stimulation and feel severe pain. It should be noted that different activities can attract attention, helping individuals feel milder pain (41). 

According to the results of the present study, children’s age significantly affected the severity of muscle vaccination pain; that is, as the age increased, the muscle vaccination pain decreased. However, Kucukoglu et al. (2016) found no significant relationship between age and the severity of muscle vaccination pain in infants (7); the difference can be due to the difference in age groups of the two studies. Infants are more sensitive to pain than other children and adults because the sensory area in infants and toddlers is the most active in the brain, and the pain transmission pathway in infants is fully developed, while the inhibitory systems are not well developed. They also have a lower pain response threshold to painful stimuli than adults and respond more quickly to painful stimuli (6). 

Failure to control all the factors involved in the intervention, e.g., the same conditions for the infants, was one of the limitations of the present study. Therefore, further studies with more control over the conditions of the infants before the intervention are suggested. Another limitation was the setting of the study, which cultural, social, or organizational factors may affect. One of the research strengths was investigating the effects of demographic characteristics on the indicator of pain severity by the analysis of the covariance test. Another strength of the present study was investigating the impact of white noise (bird sound) on vaccination pain and individual cognitive-behavioral pain variables in children for the first time worldwide. Since bird sound is an appropriate, easy, and inexpensive method to alleviate pain in children, it can reduce the severity of pain in children. According to the results, proposedly, health experts, nurses, and other healthcare staff provide the conditions to alleviate pain in children in vaccination rooms of health centers and hospitals to prevent the adverse effects of pain on children and encourage children to cooperate more in the following care measures. This method for children leads to a less painful and high-quality life, causing more natural development. Therefore, proposedly, the authorities of the Ministry of Health and Medical Education inform the health centers about the required instructions for using this simple method during vaccine injection and advise them to use this method as a care standard in vaccinating children and infants to relieve pain, facilitate acceptance, cope with the parents’ resistance, and improve the quality of health and treatment services. Proposedly, they develop and implement programs for all staff of the vaccination units and infant caregivers to retrain non-pharmacological pain relief strategies for infants. 

## In Conclusion

Pain relief is a basic need and right of all children. In this regard, painful processes should be predicted, studied, and relieved in infants. Accordingly, healthcare providers and nurses should minimize pain in children by using the relevant methods. The results of the present study showed that the use of white noise (bird sound) could reduce the severity of pain and behavioral responses (a set of leg movements, activity, crying, and consolability) in infants. These findings indicate the calming effects of the birds’ sounds during a painful procedure; therefore, this method can be harmless and inexpensive in modulating behavioral pain responses in infants during painful procedures, especially vaccination. 

## Authors Contribution

Conceptualization, A.SH. and F.M.; methodology, Z.F., and Z.J.A.; software, Y.Z. and F.M.; validation, Z.F., and Z.J.A.; formal analysis, A.SH. and F.M.; investigation, A.SH. and F.M.; resources, Z.F., and Z.J.A.; data curation, A.SH. and F.M.; writing—original draft preparation, A.SH. and F.M.; writing—review and editing, A.SH. and F.M.; visualization, Y.Z. F.M. supervision, Z.F., and Z.J.A.; project administration, Z.F., Y.Z. and Z.J.A.; funding acquisition, F.M. All authors have read and agreed to the published version of the manuscript

## Conflict of Interest

The authors declare no conflict of interest.
